# The utility of next‐generation sequencing in distinguishing between separate primary lung carcinomas and intrapulmonary metastasis: A case report

**DOI:** 10.1111/1759-7714.15423

**Published:** 2024-08-13

**Authors:** Shilpa S. Mantri, William D. Wallace, Jorge J. Nieva

**Affiliations:** ^1^ Department of Internal Medicine University of Southern California Los Angeles California USA; ^2^ Department of Pathology, Keck School of Medicine University of Southern California Los Angeles California USA; ^3^ Division of Medical Oncology University of Southern California/Norris Comprehensive Cancer Center Los Angeles California USA

**Keywords:** histopathology, intrapulmonary metastasis, lung adenocarcinoma, next‐generation sequencing, separate primary lung carcinomas

## Abstract

The distinction between separate primary lung carcinomas (SPLCs) and intrapulmonary metastases (IPMs) is crucial to accurate cancer staging. Histopathology‐based classification cannot always determine the relatedness of multiple tumors taken from the lung. Recently, next‐generation sequencing (NGS) has been used for biomarker determination, but it also has the potential to inform clonality determination among multiple tumors. Here we present a patient with three lung tumors, each diagnosed as adenocarcinoma by histopathology with a differential diagnosis of SPLC versus IPM. We pursued molecular profiling by NGS, which revealed three unique mutational patterns ruling out the possibility of clonal relatedness among the cancers. Our case supports the utility of NGS in supplementing histopathological methods to distinguish between SPLCs and IPMs and to guide treatment decisions.

## Graphical abstract

The presentation of multiple lung nodules on computed tomography scan may represent separate primary lung carcinomas or intrapulmonary metastases. Our patient was diagnosed with three separate lung adenocarcinomas, where next‐generation sequencing was required to distinguish metastases from a new primary cancer. Next‐generation sequencing may supplement histopathological methods to accurately stage disease and select appropriate treatment.

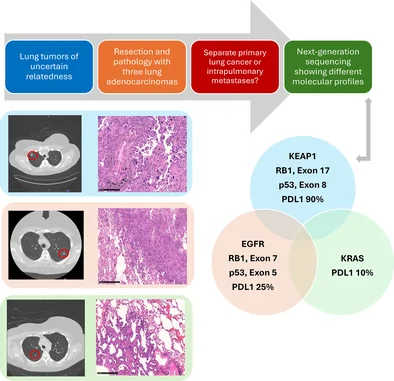

## INTRODUCTION

Advances in lung cancer detection by computed tomography (CT) scan have increased the frequency of detecting multiple pulmonary tumor nodules in clinical practice, accounting for approximately 10% of all surgically removed lung cancers.^1^, ^2^ When patients are found to have multiple primary lung lesions, determining the lineage relationship is necessary for staging disease and selecting appropriate treatment.

Patients with multiple lung nodules, particularly in cases that lack features of extrapulmonary disease dissemination, are a staging challenge for pathologists and oncologists, as they may represent separate primary lung carcinomas (SPLCs) and/or intrapulmonary metastases (IPMs) originating from a separate primary lung tumor.^3^ While SPLCs are potentially cured by surgical resection, IPMs usually require systemic therapy.^4^

In routine clinical practice, histopathology‐based classification is implemented to distinguish between SPLCs and IPMs. The current American Joint Committee on Cancer staging system recommends using comprehensive histological assessment in the evaluation of multiple primary lung tumors.^4^, ^5^ Tumors are classified as SPLCs or IPMs based on comparison of histological subtypes and cytologic and stromal features between the separate tumors. However, evaluation by histopathology is relatively subjective and affected by interobserver variability.^4^

A more informative approach would be to investigate an expanded panel of genes by next‐generation sequencing (NGS). In this report, we present a patient with three lung adenocarcinomas in which we used NGS to differentiate SPLCs from IPMs.

## CASE REPORT

**FIGURE 1:**
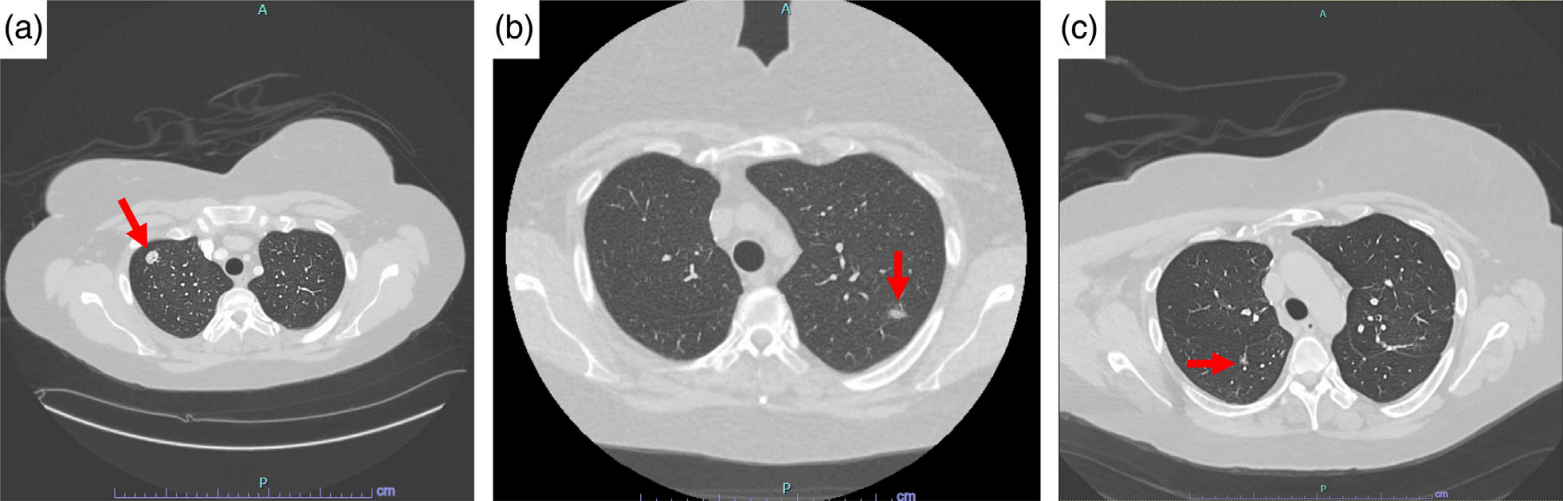
(a) A 1.3 cm pulmonary nodule (red arrow) with spiculated margins is noted along the superior aspect in the anterior periphery of the right upper lobe. The patient underwent lobectomy of this nodule. (b) A 11 × 6 mm solid pulmonary nodule (red arrow) is noted in the left upper lobe, with progressive increase in size from prior scans, while additional smaller micronodules remain grossly unchanged. The patient was treated with bisegmentectomy. (c) A 4 mm nodular density (red arrow) is noted in the right lower lung, for which the patient underwent wedge resection.

**FIGURE 2:**
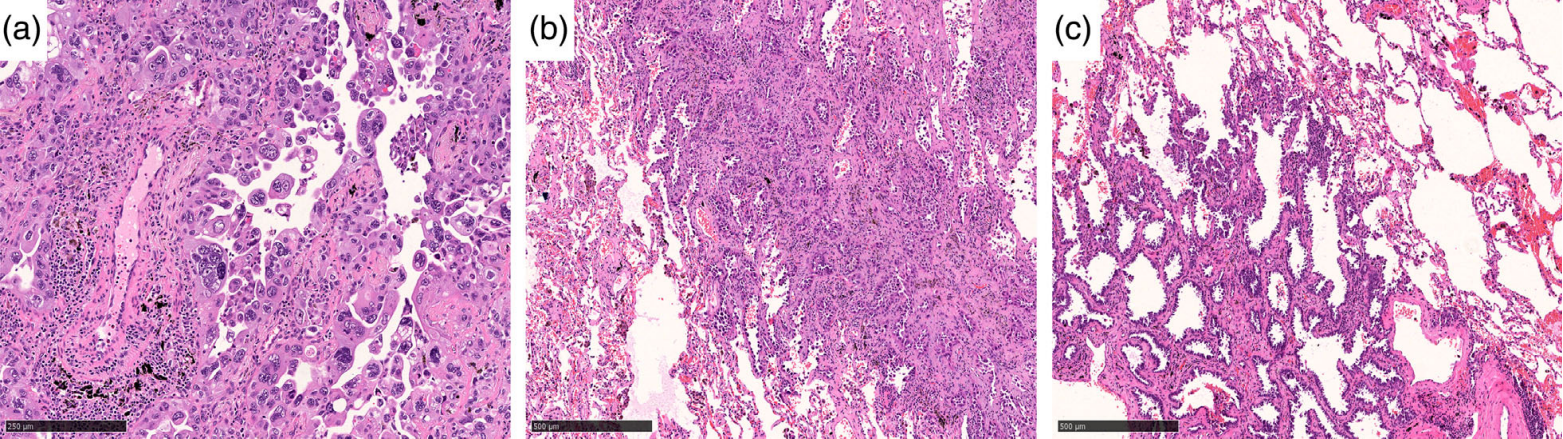
(a) Right upper lobe: The tumor is composed of invasive nests of large epithelioid cells with pleomorphic nuclei; some cells have prominent multinucleation. The tumor stroma contains infiltrating mononuclear cells with anthracotic pigment (hematoxylin and eosin stain; original magnification 100×). (b) Left upper lobe: The edge of the tumor reveals a lepidic growth pattern with pleomorphic cells lining the thickened alveolar walls. In deeper areas of the tumor, the stroma has solidified, and the tumor demonstrates glandular architecture with a mononuclear cell infiltrate in the stroma (hematoxylin and eosin stain; original magnification 50×). (c) Right lower lobe: The tumor reveals an almost entirely lepidic growth pattern, consistent with an in‐situ process. The stroma contains scattered inflammatory cells and anthracotic pigment adjacent to the larger vessels (hematoxylin and eosin stain; original magnification 50×).

A 49‐year‐old Caucasian female nonsmoker was diagnosed with left clear cell renal carcinoma and subsequently underwent a partial nephrectomy followed by annual surveillance imaging. NGS of this tumor was not performed. Five years after diagnosis, the patient was found to have a 1.3 cm right upper lobe (RUL) pulmonary nodule with spiculated margins on CT scan of the chest (Figure 1a). Following biopsy, a RUL lobectomy was performed and a pT1aN0 solid predominant lung adenocarcinoma was found (Figure 2a).

In subsequent surveillance imaging 3 years later, the patient was noted to have a new growing 11 × 6 mm left upper lobe (LUL) nodule (Figure 1b). The pathology from the subsequent bisegmentectomy specimen revealed pT1aN0 adenocarcinoma (Figure 2b). This tumor was compared histologically with the previously resected RUL tumor and due to the dissimilar pleomorphic histological features of the first tumor, the two tumors were determined to be separate primary tumors.

There were several pulmonary micronodules which were being monitored over time and 2 years later, the patient was found to have a right lower lobe (RLL) 4 mm nodule that appeared denser and larger compared with prior examinations (Figure 1c). Subsequent wedge resection of this nodule revealed pT1miN0 minimally invasive adenocarcinoma (Figure 2c). Minimally invasive adenocarcinoma is considered a primary lesion, but given the patient's history of multiple adenocarcinomas, we pursued molecular profiling by NGS to determine if this most recent tumor represented a metastasis from either of the prior tumors and to rule out a pathological interpretation error.

Direct sequence analysis was performed on genomic DNA isolated from each tumor, with 700+ clinically relevant genes analyzed. Driver mutations and relevant gene mutations were determined (Table 1). Based on NGS, all tumors had unique profiles and were unrelated to one another. Although two of the three cancers had p53 and RB1, the mutation loci were different, suggesting independent origins of these cancers.

**TABLE 1:** Staging and molecular profile of the patient's separate lung adenocarcinomas.

Lung tumor location	TNM stage	Driver mutation	Pathogenic alterations
Right upper lobe	T1aN0	*KEAP1*/exon 3, p.G332C	RB1/exon 17, p.W563* P53/exon 8, p.E298* PDL1 90%
Left upper lobe	T1aN0	*EGFR*/exon 21, p.L858R	RB1/exon 7, p.V205fs P53/exon 5, p.V157F PDL1 25%
Right lower lobe	T1miN0	*KRAS*/exon 2, p.GI2V	PDL1 10%

*Note*: The location and TNM staging of each tumor determined by histopathology are outlined. Subsequent next‐generation sequencing results for each of the patient's lung tumors demonstrate individual driver mutations with three unique mutational patterns.

Abbreviation: TNM, tumor, node, metastasis.

* Designates a stop codon.

The patient had no other known environmental exposures and underwent genetic counseling with no heritable syndromes identified. Since it was determined all three tumors were independent and stage 1, the patient did not require adjuvant therapy. The patient has had no evidence of disease on surveillance CT imaging obtained every 6 months.

## DISCUSSION

The distinction between SPLCs from IPMs in patients with multiple lung adenocarcinomas is a challenging but clinically significant issue. Our case highlights the diagnostic and therapeutic implications of distinguishing between SPLCs and IPMs. The patient had three separate pulmonary nodules, all diagnosed as adenocarcinomas, which would raise the concern of possible IPMs. Prior to the availability of sequencing data, one of the treating physicians initially staged the patient as having M1a disease and recommended systemic chemotherapy as the primary treatment modality. Although p53 and RB1 mutations were discovered in two of the three tumors, these mutations were nonsynonymous, indicating a definitive diagnosis of SPLCs.

The use of single gene panels such as separate *EGFR*, *KRAS*, *ALK*, and *BRAF* contributes to inconclusive results in approximately 20% of cases when using molecular analyses to identify only driver mutations.^4^ The identification or presence of only one common driver mutation in tumor pairs do not always allow for determination of clonal origin. The spectrum of uncommon mutations is diverse, with most of them typically seen with larger multigene NGS panels which maximize discriminatory power between SPLCs and IPMs.^4^

Several studies have compared the efficacy of NGS‐based methods in distinguishing SPLCs and IPMs with that of histopathology, overall demonstrating the superiority of NGS to traditional histopathological methods.^6^ In the largest known study comparing both methods for multiple lung tumors, Chang et al. found that 22% of tumor pairs showed discordant results between histologic prediction and final molecular classification.^7^

Other studies similarly compare interobserver agreement between different pathologists and the concordance between histological and NGS classifications. Yang et al. found that in tumor pairs where a complete agreement was not reached by different pathologists, the histological classification was concordant with the molecular classification by NGS in only 53.3% of pairs versus 100% in tumor pairs where all pathologists concurred.^4^

Given the overall accuracy of comprehensive histological assessment and the particular scenarios where discrepancies occurred, these studies suggest histological assessment may be helpful in triaging cases that would benefit from molecular confirmation.^7^ A comprehensive diagnostic approach incorporating radiological, histopathological, and molecular analyses is valuable in distinguishing SPLCs from IPMs, although large prospective studies are necessary to establish a clear approach that may be implemented in drawing such a critical distinction in clinical practice.

## AUTHOR CONTRIBUTIONS

Shilpa S. Mantri: Prepared the original draft of the manuscript. William D. Wallace: Provided analysis for the histopathological images. Jorge J. Nieva: Was responsible for obtaining the clinical data and manuscript writing/editing. All authors were involved with the conceptualization, design, and review of the final manuscript.

## CONFLICT OF INTEREST STATEMENT

The authors declare that they have no known competing financial interests or personal relationships that could have appeared to influence the work reported in this article.

## Data Availability

The datasets used and/or analyzed during the current study are available from the corresponding author upon request.
